# The global burden of kidney disease and the sustainable development goals

**DOI:** 10.2471/BLT.17.206441

**Published:** 2018-04-20

**Authors:** Valerie A Luyckx, Marcello Tonelli, John W Stanifer

**Affiliations:** a Institute of Biomedical Ethics and History of Medicine, University of Zurich, Winterthurerstrasse 30, 8006 Zurich, Switzerland.; b Department of Medicine, University of Calgary, Calgary, Canada.; c Department of Medicine, Duke University, Durham, United States of America.

## Abstract

Kidney disease has been described as the most neglected chronic disease. Reliable estimates of the global burden of kidney disease require more population-based studies, but specific risks occur across the socioeconomic spectrum from poverty to affluence, from malnutrition to obesity, in agrarian to post-industrial settings, and along the life course from newborns to older people. A range of communicable and noncommunicable diseases result in renal complications and many people who have kidney disease lack access to care. The causes, consequences and costs of kidney diseases have implications for public health policy in all countries. The risks of kidney disease are also influenced by ethnicity, gender, location and lifestyle.  Increasing economic and health disparities, migration, demographic transition, unsafe working conditions and environmental threats, natural disasters and pollution may thwart attempts to reduce the morbidity and mortality from kidney disease. A multisectoral approach is needed to tackle the global burden of kidney disease. The sustainable development goals (SDGs) emphasize the importance of a multisectoral approach to health. We map the actions towards achieving all of the SDGs that have the potential to improve understanding, measurement, prevention and treatment of kidney disease in all age groups. These actions can also foster treatment innovations and reduce the burden of such disease in future generations.

## Résumé

La maladie rénale est décrite comme la maladie chronique la plus négligée. Si d'autres études en population sont nécessaires pour établir des estimations fiables de la charge mondiale de la maladie rénale, les risques spécifiques sont présents dans l'ensemble du spectre socioéconomique, à la fois en situation de pauvreté et de richesse, de malnutrition et d'obésité, dans des environnements agricoles et postindustriels, et à tous les âges, aussi bien chez les nouveau-nés que chez les personnes âgées. Diverses maladies transmissibles et non transmissibles entraînent des complications rénales et de nombreuses personnes atteintes de maladie rénale n'ont pas accès aux soins. Les causes, les conséquences et les coûts de la maladie rénale ont une incidence sur la politique de santé publique dans tous les pays. Le risque de développer une maladie rénale est également influencé par l'origine ethnique, le sexe, le lieu et le mode de vie. L'accroissement des disparités économiques et sanitaires, les migrations, la transition démographique, les conditions de travail dangereuses, les menaces environnementales, les catastrophes naturelles et la pollution sont susceptibles de faire échouer les tentatives de réduction de la morbidité et de la mortalité liées à la maladie rénale. Une approche multisectorielle est nécessaire pour faire face à la charge mondiale de la maladie rénale. Les objectifs de développement durable (ODD) soulignent l'importance d'une approche multisectorielle en matière de santé. Nous établissons une cartographie des actions à entreprendre pour atteindre tous les ODD qui sont susceptibles d'améliorer la connaissance, la mesure, la prévention et le traitement de la maladie rénale dans toutes les tranches d'âge. Ces actions peuvent également favoriser les innovations thérapeutiques et réduire la charge de cette affection pour les générations futures.

## Resumen

La insuficiencia renal se ha descrito como la enfermedad crónica más olvidada. Serían necesarios más estudios basados en la población para obtener estimaciones fiables de la carga mundial de la insuficiencia renal, pero existen riesgos específicos en todo el espectro socioeconómico desde la pobreza hasta la prosperidad, desde la desnutrición hasta la obesidad, en contextos agrarios y postindustriales, y a lo largo de la vida desde recién nacidos hasta la tercera edad. Una variedad de enfermedades contagiosas y no contagiosas producen complicaciones renales y muchas personas que padecen una insuficiencia renal no tienen acceso a la atención. Las causas, las consecuencias y los costes de las insuficiencias renales tienen implicaciones para la política de salud pública en todos los países. Los riesgos de la insuficiencia renal también están influenciados por la raza, el sexo, la ubicación y el estilo de vida.  El aumento de las disparidades económicas y de salud, la migración, la transición demográfica, las condiciones de trabajo inseguras y las amenazas ambientales, los desastres naturales y la contaminación pueden frustrar los intentos de reducir la morbilidad y la mortalidad por insuficiencia renal. Se necesita un enfoque multisectorial para abordar la carga mundial de la insuficiencia renal. Los Objetivos de Desarrollo Sostenible (ODS) hacen hincapié en la importancia de un enfoque multisectorial de la salud. Planificamos las acciones para alcanzar todos los ODS con el potencial de mejorar la comprensión, la medición, la prevención y el tratamiento de la insuficiencia renal en todos los grupos de edad. Estas acciones también pueden fomentar innovaciones en el tratamiento y reducir la carga de dicha enfermedad en las generaciones futuras.

## ملخص

وُصف مرض الكلى بأنه المرض المزمن الأكثر عرضة للإهمال. ولا سبيل إلى الخروج بتقديرات موثوقة لحجم العبء العالمي لمرض الكلى من دون إجراء المزيد من الدراسات المستندة إلى الشرائح السكانية، إلا أن هناك مخاطر محددة تقع في مختلف أنحاء الطيف الاجتماعي الاقتصادي، ما بين الفقر إلى الثراء، ومن سوء التغذية إلى السِمنة الزائدة، وفي البيئات الزراعية إلى ما بعد الصناعية، وعلى مدار الحياة بدءًا من المولودين حديثًا حتى المسنين. وهناك عدد من الأمراض السارية وغير السارية التي تؤدي إلى إصابة الكلى بمضاعفات، ولا يتسنى للعديد من المصابين بمرض الكلى سبل الحصول على الرعاية. كما أن لمسببات أمراض الكلى وتبعاتها ونفقاتها تبعات تقع على عاتق سياسة الصحة العمومية في جميع البلدان. كما تتأثر مخاطر الإصابة بمرض الكلى بعوامل الأصل العرقي والنوع الاجتماعي والموقع ونمط الحياة. وقد يؤدي تزايد الفوارق الاقتصادية والصحية، وعوامل الهجرة، والانتقال الديموغرافي، وظروف العمل غير الآمنة، والتهديدات البيئية، والكوارث الطبيعية، والتلوث إلى تقويض المحاولات الساعية إلى الحد من نسب الإصابة والوفيات الناتجة عن مرض الكلى. ويلزم اتباع نهج متعدد القطاعات للتعامل مع العبء العالمي لمرض الكلى. وتؤكد أهداف التنمية المستدامة على أهمية اتباع نهج متعدد القطاعات للتعاطي مع الصحة. ونحن نعمل على توجيه الإجراءات نحو تحقيق جميع أهداف التنمية المستدامة القادرة على تحسين مستويات فهم مرض الكلى وقياسه والوقاية منه وعلاجه في جميع الفئات العمرية. كما يمكن لهذه الإجراءات أن تعزز من سبل التطوير في مجال العلاج، وتحد من حجم العبء الناتج عن المرض في أوساط الأجيال المقبلة.

## 摘要

肾病被视为全球最被忽视的慢性疾病。全球肾病负担的可靠预估需要更多基于人群的研究，但是特定风险的发生横跨社会各经济范围，从贫困到富裕，从营养不良到肥胖，从农耕时代到后工业时代，生命周期从新生儿到老年人。肾脏并发症导致一系列可传染和不可传染的疾病，许多患有肾病的人缺乏治疗途径。肾病的成因、结果和成本对所有国家的公众健康政策都有影响。肾病的风险也受种族、性别、地点和生活方式的影响。日益扩大的经济健康差距、移民、人口转型、不安全的工作条件和环境威胁、自然灾害与污染可能会阻碍试图降低肾病发病率和死亡率的努力。解决全球肾病负担需要多部门合作。可持续发展目标强调多部门合作解决健康问题的重要性。我们列出达成可持续发展目标所采取的措施，这些措施有提升各年龄群体对肾病理解、测量、预防和治愈的潜力。这些措施也可以促进治疗创新，减轻子孙后代的肾病负担。

## Резюме

Хроническая болезнь почек известна как одно из тех хронических заболеваний, которым уделяется меньше всего внимания. Надежные оценки глобального бремени хронической болезни почек требуют проведения исследований, более ориентированных на популяционный уровень, но определенные риски возникают во всем социально-экономическом спектре: от нищеты до богатства, от недоедания до ожирения, от аграрного до постиндустриального сегментов общества, а также в течение всей жизни от новорожденных до взрослых людей. Ряд инфекционных и неинфекционных заболеваний приводит к осложнениям, затрагивающим почки, а многие люди с хронической болезнью почек не имеют возможности получить медицинскую помощь. Причины, последствия и расходы, связанные с заболеваниями почек, имеют значение для политики общественного здравоохранения во всех странах. Риск развития хронической болезни почек также зависит от этнической принадлежности, пола, территории проживания и образа жизни.  Рост диспропорций в области экономики и здравоохранения, миграция населения, демографические изменения, небезопасные условия труда и экологические угрозы, стихийные бедствия и природное загрязнение могут помешать попыткам снизить заболеваемость хронической болезнью почек и связанную с ней смертность. Для решения проблемы глобального бремени хронической болезни почек необходим многосекторальный подход. Цели в области устойчивого развития подчеркивают важность многосекторального подхода к здравоохранению. Авторы составили план действий по достижению всех целей в области устойчивого развития, которые могут улучшить понимание аспектов хронической болезни почек во всех возрастных группах, а также исследование, профилактику и лечение этой болезни. Эти действия могут также способствовать инновациям в области лечения и уменьшить бремя этого заболевания для будущих поколений.

## Introduction

The 17 sustainable development goals (SDGs) were adopted by the United Nations, as successors to the millennium development goals, with the broad goal of achieving healthy people living on a healthy planet.^1^ Although only SDG 3, that is, to ensure healthy lives and promote well-being for all at all ages, is specifically focused on health,^1^ achievement of all of the SDGs should have health benefits via impacts on the environment, governance and society.

The *Global action plan for the prevention and control of noncommunicable diseases 2013–2020* (hereafter called the 2013 action plan) outlined an approach to reduce the combined mortality from four major categories of noncommunicable disease, i.e. cancer, cardiovascular disease, chronic respiratory disease and diabetes, by 25% by 2025.^2^ Previously, these four categories had been prioritized in the 2008–2013 action plan because, collectively, they were believed to account for about 60% of global deaths and it was anticipated that a large proportion of these deaths could be prevented through elimination of shared risk factors, e.g. alcohol and tobacco use, poor diets and inadequate exercise.^3^ Although laudable, the 2013 action plan has been criticized for failing to acknowledge the broader drivers of the noncommunicable disease epidemics, other important noncommunicable diseases and the so-called causes of the causes of noncommunicable diseases and failing to place sufficient emphasis on the need for coordinated multisectoral action.^4^ We argue that kidney disease represents one of the important noncommunicable diseases missing from the 2013 action plan and that, given the many social and structural factors that directly affect risks and outcomes of kidney disease, multisectoral action to achieve the SDGs will help prevent and control such disease (Table 1).^1^

**Table 1:** The 17 sustainable development goals and their relevance to kidney health, 2015

Goal	Description	Relevance to kidney health	Relevant SDG 3 targets
1	End poverty in all its forms everywhere	Improvements in access to nutrition, personal safety and health care should enhance the prevention, detection and management of kidney disease Should reduce the incidence of catastrophic health expenditure resulting from treatment for kidney disease	3.8
2	End hunger, achieve food security and improved nutrition and promote sustainable agriculture	Improvements in maternal nutrition and reductions in the frequencies of low birth weight and preterm birth should reduce the risk of CKD Reductions in the incidence of obesity should cut the risk of CKD, diabetes and hypertension	3.1, 3.2
3	Ensure healthy lives and promote well-being for all at all ages	Should improve screening for, and the prevention, diagnosis and treatment of, kidney disease Public health programmes to promote community education, healthy lifestyles and vaccinations could also reduce the risk of AKI and CKD	All
4	Ensure inclusive and equitable quality education for all and promote life-long learning	Should improve awareness and kidney-health-related knowledge May reduce use of nephrotoxic remedies and preparations	3.4, 3.5
5	Achieve gender equality and empower women and girls	Reductions in the numbers of teenage pregnancies and increases in pregnancy spacing may reduce the incidence of the low birth weight, prematurity and pregnancy-related complications that are all risk factors for CKD There should also be improvements in overall family health	3.1, 3.7
6	Ensure access to water and sanitation for all	There should be reductions in the incidence of the waterborne diseases and diarrhoeal illnesses that are major causes of AKI and in the incidence of the schistosomiasis that can cause CKD There should also be reductions in water pollution that can cause CKD	3.9
7	Ensure access to affordable, reliable, sustainable and modern energy for all	Should broaden opportunities to use mobile health in prevention and treatment and in community and health worker education Improvements in access to electronic information sharing and data collection could lead to improvements in the epidemiology, monitoring and surveillance of kidney disease	3.9
8	Promote inclusive and sustainable economic growth, employment and decent work for all	Improvements in personal access to health care, dignity and wealth could lead to improvements in the prevention and early treatment of kidney disease Improvements in the retention of health-care workers could reduce the so-called brain drain Task shifting in health care could be facilitated	3.b, 3.c
9	Build resilient infrastructure, promote sustainable industrialization and foster innovation	Could support innovations to improve the affordability and sustainability of access to diagnosis, facilitate early treatment and secondary prevention and foster cheaper and more efficient means to prevent, diagnose and treat both AKI and CKD Could also facilitate investigation of the potential benefits of, or risks posed by, traditional remedies for kidney disease	3.b
10	Reduce inequality within and among countries	Could improve equity in the prevention, diagnosis and care of all forms of kidney disease Could improve access to expensive therapies, e.g. dialysis, hepatitis C therapy and transplantation Could improve geographical access to all forms of kidney care	3.1, 3.2, 3.7, 3.8, 3.b, 3.d
11	Make cities inclusive, safe, resilient and sustainable	Improved warning and protection from disasters could reduce crush-injury-related AKI Levels of preparedness in mass disasters, including for patients with AKI, CKD or ESKD, should improve Urban planning to eliminate food deserts and increase physical activity could help reduce diabetes and obesity-related kidney disease Reductions in exposure to alcohol, drugs and tobacco could also reduce the risk of kidney disease	3.5, 3.6, 3.d
12	Ensure sustainable consumption and production patterns	Promotion of the environmentally friendly and sustainable local production of dialysis supplies could reduce dialysis costs, create jobs and support the local economy Any reductions in the need for dialysis should reduce the carbon footprint from dialysis There may also be adverse effects on kidney health as increasing access to cars and unhealthy processed foods could lead to an increasing prevalence of obesity and access to cigarettes may also increase	3.4, 3.5, 3.9
13	Take urgent action to combat climate change and its impacts	Global warming may have contributed to an epidemic of Central American nephropathy and to CKD of unknown origin that appears related to dehydration and toxin exposure The adverse effects of climate change on the transmission of pathogens causing infectious disease and poverty may increase the risk of CKD	3.2, 3.3, 3.d
14	Conserve and sustainably use the oceans, seas and marine resources	Exposure to marine pollution may increase the risk of CKD	3.9, 3.d
15	Sustainably manage forests, combat desertification and halt and reverse land degradation and halt biodiversity loss	Any reduction in the leaching of toxins from industrial waste into ground water could reduce the risk of the CKD associated with such pollution	3.9, 3.d
16	Promote just, peaceful and inclusive societies	Any reduction in armed conflict could reduce the risk of AKI associated with crush injuries and major trauma and improve food security The incidence of low birth weight, which is a risk factor for CKD, tends to increase during wars Among prisoners and other marginalized populations, improvements in equity and justice could facilitate the prevention, diagnosis and treatment of kidney disease	3.d
17	Revitalize the global partnership for sustainable development	Improved global partnerships for health-care financing and regulation and health-related development and research could accelerate our understanding of kidney disease, reduce inequities in kidney care and reduce so-called transplant tourism	3.d

AKI: acute kidney injury; CKD: chronic kidney disease; ESKD: end-stage kidney disease; SDG: sustainable development goal.

## Global burden

Although often considered a comorbidity of diabetes or hypertension, kidney disease has numerous complex causes.^5^ Importantly, such disease has an indirect impact on global morbidity and mortality by increasing the risks associated with at least five other major killers: cardiovascular diseases, diabetes, hypertension, infection with human immunodeficiency virus (HIV) and malaria. For example, the Global Burden of Disease (GBD) 2015 study estimated that 1.2 million deaths, 19 million disability-adjusted life-years (DALYs) and 18 million years of life lost from cardiovascular diseases were directly attributable to reduced glomerular filtration rates.^6^^,^^7^

The GBD 2015 study also estimated that, in 2015, 1.2 million people died from kidney failure, an increase of 32% since 2005.^7^ In 2010, an estimated 2.3–7.1 million people with end-stage kidney disease died without access to chronic dialysis.^8^ Additionally, each year, around 1.7 million people are thought to die from acute kidney injury.^9^ Overall, therefore, an estimated 5–10 million people die annually from kidney disease. Given the limited epidemiological data, the common lack of awareness and the frequently poor access to laboratory services, such numbers probably underestimate the true burden posed by kidney disease. It is therefore possible that, each year, at least as many deaths are attributable to kidney disease as to cancer, diabetes or respiratory diseases, three of the four main categories targeted by the 2013 action plan.^2^^,^^10^^,^^11^ In addition, the estimated number of DALYS attributable to kidney disease globally increased from 19 million in 1990 to 33 million in 2013.^12^ In 2016, the DALYs associated with chronic kidney disease, along with those associated with cardiovascular disease, cancers, diabetes and neurological disorders, were found to have increased significantly between 1990 and 2015.^6^ A report from the GBD 2016 study highlighted the important omission of focus on chronic kidney disease and suggested that “the SDG agenda offers at best a minimal platform for drawing attention to the health care and monitoring needs of [chronic kidney disease].”^13^

Kidney disease is associated with a tremendous economic burden. High-income countries typically spend more than 2–3% of their annual health-care budget on the treatment of end-stage kidney disease, even though those receiving such treatment represent under 0.03% of the total population.^14^ In 2010, 2.62 million people received dialysis worldwide and the need for dialysis was projected to double by 2030.^8^ Globally, the total cost of the treatment of the milder forms of chronic kidney disease appears to be much greater than the total cost of treating end-stage kidney disease. In 2015, in the United States of America, for example, Medicare expenditures on chronic and end-stage kidney disease were more than 64 billion and 34 billion United States dollars, respectively.^15^ Much of the expenditure, morbidity and mortality previously attributed to diabetes and hypertension are attributable to kidney disease and its complications.^12^^,^^16^

Worldwide, important risk factors for kidney disease include diarrhoeal diseases, HIV infection, low birth weight, malaria and preterm birth, all of which are also leading global causes of DALYs.^12^ Risks of kidney disease span the life-course and environmental, infection and lifestyle etiologies.^17^ If risk factors are identified early, acute kidney injury and chronic kidney disease can be prevented and, if kidney disease is diagnosed early, worsening of kidney function can be slowed or averted by inexpensive interventions, several of which are on the World Health Organization’s (WHO’s) so-called best buys list for noncommunicable disease management.^18^ Such interventions include counselling for cardiovascular disease, diabetes and hypertension, drug therapy, tobacco control, promotion of physical activity and the reduction of salt intake through legislation and food labelling. The timely identification and management of acute kidney injury and chronic kidney disease represent the most effective strategy to address the growing global burden sustainably.^4^^,^^5^ By advocating for a multisectoral approach, as a means to achieving the SDGs, it should be possible to reduce the incidence of kidney disease globally.^19^ We discuss the kidney-health-related opportunities offered by attempts to achieve each SDG (Table 1).

## SDGs and kidney health

### SDGs 1, 3.8, 3.b and 10

In high-income countries, lower socioeconomic status is associated with greater risk of end-stage kidney disease because of behavioural and metabolic risk factors and reduced access to care.^20^ In low- and middle-income countries, the burden posed by such poverty-related kidney disease is even greater, because of associated infections, hazardous work, poor education and poor maternal health. In all countries, poverty is associated with lack of social protection and transportation, poor housing and unemployment.^20^ Lack of transportation restricts access to care even when treatment costs are not a major barrier.^20^ Poverty and lower socioeconomic status have been specifically identified as independent risks for both incident chronic kidney disease and the more rapid progression of such disease.^20^ In low-income countries where treatment costs have to be paid directly by patients, a month’s supply of essential medications for the treatment of chronic kidney disease can cost up to 18 days’ wages^21^ and the corresponding out-of-pocket costs of dialysis, for acute kidney injury or end-stage kidney disease, are much higher.^22^^,^^23^ In South Africa, where limited access to dialysis is government-funded, patients who are otherwise eligible for dialysis are frequently declined access because of their socioeconomic circumstances.^24^ For those who do access dialysis, the financial burden is exacerbated because they cannot be employed while receiving dialysis or travelling to and from the provider.

Promotion of universal health coverage should reduce the financial hardship of patients with kidney disease and improve access to kidney care.^25^ The goal of eradicating poverty spans all of the other SDGs and is fundamental to improving kidney health. In turn, achievement of each SDG promises to promote equity and reduce poverty.^20^

### SDG 2

Many low-income countries have problems with undernutrition and overnutrition, both are risk factors for kidney disease. Malnutrition predisposes young children to infections, e.g. diarrhoeal diseases and pneumonia, that are important risk factors for acute kidney injury.^22^ Among girls and female adolescents, undernutrition leads to underweight mothers and low-birth-weight offspring.^26^ Low birth weights, preterm births and pregnancies affected by diabetes and pre-eclampsia, which, combined, may represent up to 20% of pregnancies worldwide, are all associated with increased lifetime risk of chronic kidney disease in both mothers and children.^26^ Obesity increases the lifetime risk of end-stage kidney disease^17^ and maternal obesity is associated with adverse outcomes in pregnancy,^26^ including the gestational diabetes and preterm births that are associated with increased risk of chronic kidney disease.

Adequate nutrition is a key tool for reducing the burden of chronic kidney disease. Groups with very low incomes often live in areas where access to healthful foods is very limited or non-existent.^20^ Some population-level strategies, e.g. public education about healthful food choices, regulation of the fat, salt and/or sugar contents of food and the regulation of programmes for the provision of public and/or school meals, can all improve kidney health.^27^ Reduction in dietary salt is proposed as a cost-saving best buy with great potential to avert deaths from kidney disease. Similarly, a tax on high-sugar beverages, as introduced in Mexico, where chronic kidney disease is the second leading cause of death, can lead to sustained decreases in the purchase of taxed drinks and may reduce diabetes-related kidney disease over time.^28^

### SDG 3

SDG 3 has many links to better kidney health (Table 2 available at: http://www.who.int/bulletin/volumes/96/6/17-206441) including optimization of fetal development, prevention of infections, reduction of the mortality and morbidity of cardiovascular disease and mitigation of environmental exposures. The Global Kidney Health Atlas has provided an overview of the main gaps in kidney care globally: an absence of relevant policies, shortages of essential medications, reliable epidemiological data, relevant workforce capacity, infrastructure and research capacity and a persistent reliance on out-of-pocket payments.^29^ The Atlas emphasizes the need for a health-system-wide approach to kidney care and provides a baseline against which to measure progress. Work towards reducing the global burden of kidney disease will contribute to achieving SDG 3 (Table 2).

**Table 2:** Relevance of the targets of sustainable development goal 3 to kidney disease, 2015

Target	Description	Relevant kidney condition	Strategies or actions to reduce risk of kidney disease	Policies facilitating improved kidney health
3.1	By 2030, reduce global maternal mortality to less than 70 deaths per 100 000 live births	Pregnancy-related AKI and pre-eclampsia	Improve access to antenatal care and institutional deliveries and the recognition of pregnancy complications, e.g. eclampsia, pre-eclampsia and peripartum haemorrhage	UHC Promotion of gender equity Public health promotion of healthy lifestyles through education and regulation of unhealthy food and tobacco consumption Promotion of the consumption of healthy food
			Identify, during antenatal care or at delivery, mothers at risk, for education and follow-up to reduce long-term risk of maternal CKD and cardiovascular disease associated with pre-eclampsia	
3.2	By 2030, end preventable deaths of neonates and children under 5 years of age, with all countries aiming to reduce neonatal mortality to no more than 12 deaths per 1000 live births and mortality among children under 5 years of age to no more than 25 deaths per 1000 live births	Perinatal AKI	Reduce prematurity	UHC Education of health-care workers Enhancement of the capacity and infrastructure for detection and surveillance
			Avoid or reduce perinatal use of nephrotoxins, e.g. aminoglycoside antibiotics and non-steroidal anti-inflammatory drugs	
			Optimize neonatal nutrition	
		CKD and hypertension in later life	Reduce prematurity and low birth weight, which are both risk factors for low nephron numbers	UHC to improve access to prevention and screening services Public health promotion of healthy lifestyles through education and regulation of unhealthy food and tobacco consumption Adoption and implementation of the FCTC
		Diarrhoea-associated and HUS-associated AKI, post-infectious glomerulonephritis	Improve sanitation and access to vaccinations and medical care	Development of public health policy to improve disease surveillance and response to outbreaks
3.3	By 2030, end the epidemics of AIDS, malaria, neglected tropical diseases and tuberculosis, and combat hepatitis, waterborne diseases and other communicable diseases	HIV nephropathy	Provide equitable access to services for the prevention and treatment of HIV infection	UHC Enactment of protections for women victims of domestic violence and sexual assault Taking action, including legal, policy and regulatory reforms, to ensure full political enfranchisement for women Legislation for the protection of sex workers
		Malaria-associated AKI, black water fever	Prevent and provide early treatment of malaria and combat both availability of fake medication and emergence of resistance to antimalarials	Development of public health policy to improve disease surveillance and response to outbreaks Reforming of pharmaceutical supply chains and enhancement of regulations to combat fake medicines
		CKD – a risk factor for tuberculosis	Increase awareness of risk	Development of public health policy to improve disease surveillance and the effectiveness of diagnosis and treatment Development of innovative interventions to improve labour conditions and conditions in prisons
			Adapt medication doses according to kidney dysfunction	
		Hepatitis-associated glomerulonephritis and hepatorenal syndrome	Improve access to vaccination and treatment for hepatitis B and C	Provision of public education and UHC Development of care models integrating traditional healers. Legislation on alcohol consumption to reduce high-risk drinking
			Reduce hepatitis-associated inflammation and immune-complex deposition	
			Reduce kidney-disease-associated cirrhosis and liver failure	
		CKD from infections	Prevent and treat Hantavirus, leptospirosis and scrub typhus	Development of public health policy to improve disease surveillance and the effectiveness of diagnosis and treatment
		Urinary obstruction	Reduce schistosomiasis	Development of public health policy to improve disease surveillance and response to outbreaks
			Diagnose and treat kidney tuberculosis adequately, to reduce long-term obstruction of urinary tract	
3.4	By 2030, reduce by one third premature mortality from noncommunicable diseases through prevention and treatment and promote mental health and well-being	CKD	Prevent and screen for CKD, improve access to early diagnosis and effective treatment for CKD, provide equitable access to treatment for kidney failure, i.e. dialysis and transplantation, and strengthen access to options for lifestyle improvement	UHC Enactment of protections for women victims of domestic violence and sexual assault Taking action, including legal, policy and regulatory reforms, to ensure full political enfranchisement for women Promotion of healthy lifestyles through education and regulation of unhealthy food consumption Adoption and implementation of FCTC Enhancement of capacity and infrastructure for detection and surveillance Development of care models integrating traditional healers. Enhancement of occupational safety standards Development of transparent policies governing access to expensive therapies such as dialysis and transplantation
			Reduction in CKD could reduce morbidity and mortality associated with some other diseases, e.g. cancer, diabetes and liver disease	
		Cardiovascular disease	Reduce CKD, this should reduce the burdens posed by global hypertension and cardiovascular disease and the associated mortality	
		AKI	Prevent AKI through improved access to sanitation and vaccination, decrease reliance on toxic traditional remedies, improve access to early diagnosis and effective treatment for AKI and provide equitable access to dialysis	
			Reduction in AKI could reduce morbidity and mortality associated with some other conditions, e.g. heart failure, liver disease, sepsis and surgery	
3.5	Strengthen the prevention and treatment of substance abuse, including narcotic drug abuse and harmful use of alcohol	CKD and hypertension in later life	Reduce low birth weight associated with alcohol use, smoking and substance abuse in pregnancy	UHC Enactment of protection for women victims of domestic violence and sexual assault Taking action, including legal, policy and regulatory policy reforms, to ensure full political enfranchisement for women Promotion of urban safety Legislation and regulation of alcohol consumption Adoption and implementation of FCTC Legislation for the protection of sex workers
			Alcohol use and smoking are risk factors for CKD progression	
		HIV and hepatitis-associated kidney disease, infectious glomerulonephritis	Reduce infections transmitted by intravenous drug use	
		Rhabdomyolysis	Prevent rhabdomyolysis by increasing awareness and providing treatment for drug withdrawal and delirium tremens	
3.6	By 2020, halve the number of global deaths and injuries from road traffic accidents	AKI	Prevent trauma-related crush injury or blunt kidney trauma	Enforcement of existing traffic laws and reform of traffic laws to reduce road trauma Promotion of occupational safety Development and building of infrastructure and safe roads, with capacity to absorb urban growth
		CKD	Prevent accident-related losses in employment, increases in poverty and reductions in access to health care	
3.7	By 2030, ensure universal access to sexual and reproductive health-care services, including for family planning, information and education, and the integration of reproductive health into national strategies and programmes	Pregnancy-related AKI, CKD	Empower women, increase spacing of pregnancies and reduce teenage pregnancies	Promotion of access to education for all and family planning, gender equity and UHC Strengthen legislation on access to safe abortion and the protection of sex workers
			Reduce risk of low birth weight and preterm birth, as these can adversely affect kidney health of the child	
3.8	Achieve UHC, including financial risk protection, access to quality essential health-care services and access to safe, effective, quality and affordable essential medicines and vaccines for all	AKI, CKD	Provide universal access to basic health care and services for the diagnosis, prevention and treatment of all kidney disease and its risk factors, e.g. diabetes and hypertension	Promotion of innovative financing, regulation of the pricing of medical products and UHC Monitoring of catastrophic health expenditure
3.9	By 2030, substantially reduce the number of deaths and illnesses from hazardous chemicals and air, water and soil pollution and contamination	CKD of unknown origin, observed in Egypt, India and Sri Lanka, and Balkan nephropathy	Reduce exposure to environmental toxins that may be associated with CKD, e.g. aristolochic acid and cadmium and others	Promotion of environmental protection and safety Promotion of sustainable agriculture and fishing Commitment to combat climate change
3.a	Strengthen the implementation of WHO’s FCTC in all countries, as appropriate	CKD	Reduce tobacco smoking, a risk factor for cardiovascular disease and mortality, haematuria, low birth weight and proteinuria	Adoption and implementation of FCTC
3.b	Support the research and development of vaccines and medicines for the communicable and noncommunicable diseases that primarily affect developing countries, provide access to affordable essential medicines and vaccines, in accordance with the Doha Declaration on the TRIPS Agreement and Public Health, which affirms the right of developing countries to use to the full, the provisions in the TRIPS Agreement regarding flexibilities to protect public health and, in particular, provide access to medicines for all	AKI	Provide and support the uptake of vaccines that can prevent diarrhoeal illness, sepsis and other infections that can cause AKI and can prevent low birth weight in pregnancy	Promotion of budget allocation for locally relevant research Strengthening and empowerment of local research ethics committees Utilization of TRIPS Agreement exemptions Enhancement of the regulation of generic medication Monitoring of medication supply and use Promotion of health technology assessments Development of transparent policies governing access to expensive therapies, e.g. dialysis and transplantation Development of innovative financing models to reduce costs of dialysis and transplantation Implementation and enforcement of the Istanbul Declaration against organ trafficking Development of legislation regarding brain death and organ donation Opt-out or presumed-consent policies for organ donation
			Support prompt access to the intravenous fluid and appropriate antibiotics that can prevent AKI and glomerulonephritis^d^	
			Vaccination in pregnancy can reduce the risk of low birth weight	
			Vaccination during pregnancy can reduce the incidence of low birth weight	
		CKD	Provide affordable and sustainable access to basic medications for CKD, diabetes and hypertension and so reduce burden of end-stage kidney disease	
		ESKD	Devise innovative ways to deliver cheaper dialysis worldwide	
		Transplantation	Promote safe and altruistic kidney donation by living donors. Improve supply from deceased donors where permissible. Stop organ trafficking	
3.c	Substantially increase health financing and the recruitment, development, training and retention of the health workforce in developing countries, especially in least developed countries and small island developing states	Kidney disease awareness and capacity to treat	Improve awareness and capacity to diagnose, prevent and treat kidney disease	Development of innovative financing models to reduce costs of dialysis and transplantation Promotion of the education, licensing and registration of health-care workers and researchers Promotion of the fair remuneration of health-care workers Legislation to define the scope of practice of community health workers and any associated task shifting
			Train and retain health-care workers with knowledge of kidney disease	
3.d	Strengthen the capacity of all countries, in particular developing countries, for early warning, risk reduction and management of national and global health risks	Crush syndrome	Improve disaster planning and responses to earthquakes and other major disasters	Promotion of international collaboration to respond to natural disasters Commitment to equality and peace Promotion of democracy Strengthening of intersectoral communication and collaboration
		CKD	Promote peace	
			Prevention of wars should reduce both the burden of kidney disease associated with low birth weight and malnutrition and the conflict-related disruption of care	

AIDS: acquired immunodeficiency syndrome; AKI: acute kidney injury; CKD: chronic kidney disease; ESKD; end-stage kidney disease; FCTC: Framework Convention on Tobacco Control; HIV: human immunodeficiency virus; HUS: haemolytic uraemic syndrome; TRIPS: Trade-related Aspects of Intellectual Property Rights; UHC: universal health coverage; WHO: World Health Organization.

### SDGs 4 and 5

Because they are, in general, responsible for most child care and housework, women in low- and middle-income countries may face greater challenges if they have chronic kidney disease – and other noncommunicable diseases, than men with similar health problems.^30^ Heavy demands on their time may explain why, even though chronic kidney disease is more common among women than men, fewer women than men receive dialysis.^30^ Child marriage and lack of access to family planning contribute to poor maternal health and increased risk of obstetrical complications, including acute kidney injury.^31^ Among urban adults in the United States, both gender and race appeared to affect glomerular filtration rates.^32^ Achievement of equity for women worldwide should reduce the burden of kidney disease.

### SDG 6

Globally, almost 800 million people lack access to safe water and 2.5 billion lack access to optimal sanitation.^33^ In low- and middle-income countries, waterborne and pestilent diseases associated with poor hygiene and sanitation are major causes of acute kidney injury and chronic kidney disease.^34^ Enteric diarrhoeal deaths, associated with lack of safe water, cause over 1 million deaths annually.^13^ Most of these deaths occur in children younger than five years and many can be attributed to dehydration-related acute kidney injury.^13^ Non-enteric diseases caused by waterborne pathogens, e.g. leptospirosis and schistosomiasis, are also major causes of kidney disease in low- and middle-income countries.^34^

Local availability of clean water would be expected to reduce the risk of diarrhoea-related acute kidney injury.^35^ Beyond infection-related kidney complications from contaminated water and poor sanitation, additional challenges exist. Water containing organic perfluoroalkyl acids and heavy metals has been associated with chronic kidney disease in several settings and pesticide-contaminated well water may contribute to the risk of some chronic kidney disease observed in Sri Lanka.^36^ Dehydration, in conjunction with heat stress, may have contributed to the epidemic of chronic kidney disease observed among young, economically productive male labourers in Central America and South-East Asia.^36^ The global burden of kidney disease should be reduced by ensuring the availability of clean water and adequate sanitation.

### SDGs 7 and 12–15

Climate change, degradation of biodiversity, forest and land, and loss of marine resources, all likely increase the risk of kidney disease through multiple mechanisms, e.g. increases in food insecurity, the incidences of heat-related illness and infectious diseases and pollution.^37^ Deforestation and land degradation can bring humans into greater contact with vector-borne and waterborne pathogens, such as enteric bacteria and other pathogens that can directly cause kidney disease, e.g. those causing dengue fever, leishmaniasis, leptospirosis, malaria, schistosomiasis, trypanosomiasis and yellow fever.^38^

Reducing the global burden of kidney disease in turn will also be critical for mitigating some of the environmental impacts of dialysis. Each year, for example, the haemodialysis given to more than 2 million people requires 160 billion litres of water and generates over 900 000 tonnes of, predominantly plastic waste.^39^ Clean, local production of dialysis supplies, the reprocessing of dialysis filters, the reuse of dialysis water, solar-powered dialysis and waterless dialysis are all promising strategies that could creduce the environmental footprint of dialysis as well at its costs.^39^

### SDGs 8, 10 and 17

Within low- and middle-income countries, access to dialysis is highly inequitable.^8^ Despite its relative cost–effectiveness, access to transplantation is even more inequitable because of cultural, financial and legislative barriers and infrastructural limitations.^40^ In the face of extreme social inequalities and a demand for transplants that markedly exceeds the supply, the trafficking of kidneys and other human organs remains a major concern.^40^

Disparities in the burden of kidney disease, which are particularly complex, arise from biological, environmental, genetic, lifestyle and sociocultural factors^20^ and need to be addressed via multilevel, systematic interventions.^34^ An example of the complexities involved has been described in the United States. There, in general, compared with other patients with similar disease, patients with chronic kidney disease from ethnic and racial minorities have delayed referral for care, lower incomes, report poorer physician–patient relationships and have less access to health care in general.^20^ The pervasive disparities in kidney disease will have to be addressed before SDGs 8, 10 and 17 can be achieved.

### SDGs 3.6, 3.d, 9 and 11

Rapidly occurring urbanization has contributed to the rise of kidney disease and other noncommunicable diseases in low- and middle-income countries.^41^ In addition to the commonly associated lifestyle changes, e.g. a switch to high-calorie, sodium-rich diets and decreased physical activity, rapid urbanization has led to crowded cities with environmental pollution, a limited infrastructure and poor levels of sanitation and waste disposal.^42^ Such urbanization also means that more and more people are living in settings where a growing prevalence of noncommunicable diseases, e.g. diabetes, hypertension and obesity, is juxtaposed with environmental toxins and numerous infectious diseases.^42^ These changes portend a synergistic growth in the global burden of kidney disease. There may already be evidence of such growth in the ever-higher ranking of chronic kidney disease among leading cause of deaths, across all country income categories, between 1990 and 2016.^13^

By building resilient infrastructure while promoting sustainable industrialization, it should be possible to enhance health-care access while simultaneously reducing the risk of kidney disease. In low- and middle-income countries, urban planning, to improve hygiene and sanitation and reduce population densities and the transmission of the pathogens causing enteric infections, schistosomiasis and tuberculosis, should reduce the incidence of acute kidney injury and chronic kidney disease.^17^ At the same time, by promoting the development of parks, paths and efficient transport systems, urban planning could increase general levels of physical activity and so help reduce the risk of obesity-related kidney disease.^42^

The effective prevention of chronic kidney disease will require engagement with the corporate sector, whose interests may be in conflict with those of public health.^43^ Novel strategies are required to create incentives for the corporate sector to promote public health.^44^ Even under optimal circumstances, kidney disease cannot always be prevented and strategies to reduce the economic, physical and social burdens of end-stage kidney disease are needed. Innovative mechanisms to reduce dialysis costs and make dialysis less dependent on electricity and water could multiply opportunities for access to dialysis, especially in low- and middle-income countries.^39^ Innovation is also required to improve access to transplantation. Although opt-out or presumed-consent strategies have been proposed as a way of increasing the supply of organs from deceased donors, they remain contentious.

Acute kidney injury after a road-traffic collision may result from rhabdomyolysis and multi-organ failure as well as blunt or penetrating kidney injury.^45^ Natural disasters are associated with increased rates of crush-injury-induced acute kidney injury and frequently lead to life-threatening interruptions of treatment among those with end-stage kidney disease.^46^ Similarly, forced migrants with chronic or end-stage kidney disease can face dangerous interruptions in their treatment or receive inadequate care,^47^ even in a high-income country such as the United States.^48^ Continued action on reducing the burden of road-traffic injuries and supporting efforts to integrate noncommunicable disease management into humanitarian relief efforts should help to reduce the burden of chronic and end-stage kidney disease.^46^

### SDG 16

Exposure to armed conflict can result in acute kidney injury caused by crush injury and rhabdomyolysis and the severity of injuries sustained in combat strongly correlates with the subsequent risk of chronic kidney disease.^49^ Kidney disease is common in incarcerated populations and, in terms of their kidney health, prisoners may face a triple burden: of excess risk of kidney disease and its risk factors, of barriers to preventive care for established chronic kidney disease and of the suboptimal management of end-stage kidney disease.^50^ As an important step towards improving global health, much work is required globally to reduce conflict and disparities and enhance peace.

## Policy perspective

The net health burden of kidney disease is substantial, growing and driven by complex interactions, between communicable and noncommunicable diseases, that are shaped by upstream environmental and socioeconomic disparities. Although kidney disease, whether acute, chronic or end-stage, can be extremely costly, it is also potentially preventable and adverse outcomes can often be delayed or prevented by inexpensive interventions. Kidney disease is highly prevalent, spans the life course and has substantial financial implications. Our response to such disease requires a systematic policy approach, to strengthen all relevant aspects of the health system and to facilitate integration of the promotion of kidney health within a comprehensive horizontal programme for the prevention and treatment of noncommunicable diseases (Table 2).

Within each country, the local burden and prevalence of kidney disease and its risk factors and the local capacity to identify and manage such disease must be determined, as a prerequisite for fair priority setting and appropriate policy development. Diagnosis of kidney disease is often hampered by a lack of awareness among health-care workers and at-risk communities and by inadequate and often erratic access to laboratory testing. Broad policies are increasingly being adopted globally to curb dietary intakes of fat, salt and sugar. Such policies all promise to reduce the burden of chronic kidney disease. The burden of acute kidney injury could be reduced through the ongoing commitment to reduce the transmission of the pathogens causing infectious diseases.

We need universal health coverage to tackle kidney disease successfully and ensure effective screening, prevention and early treatment. Effective and transparent policies to govern access to care for end-stage kidney disease should only be developed after there has been a thorough attempt to determine the local health priorities, especially in resource-poor settings. Engagement with all relevant stakeholders and innovative financing strategies will be required to maximize equitable access to care. The bidirectional and synergistic interplay between kidney disease and all of the SGDs must be acknowledged in the development of a multisectoral approach. Policies that foster domestic and international collaboration, improve occupational and road safety, limit organ trafficking, promote access to education and gender equality, reduce unemployment and tackle the predicted adverse effects of climate change may all reduce kidney disease and/or the disparities in the care for such disease. However, as noted by the United Nations Secretary-General in December 2017, in the control and prevention of noncommunicable diseases, “political commitments have not often been translated into concrete action.”^51^ On its own, policy-making is insufficient. Monitoring the impact of policies on kidney disease and the risk factors for such disease needs to be integrated into existing surveillance activities. Health workers and communities must be empowered to advocate for, and hold policy-makers accountable for, kidney health, as an important step towards achievement of the SDGs.
